# PET Stress Testing with Coronary Flow Capacity in the Evaluation of Patients with Coronary Artery Disease and Left Ventricular Dysfunction: Rethinking the Current Paradigm

**DOI:** 10.1007/s11886-021-01478-3

**Published:** 2021-03-24

**Authors:** Robert M. Bober, Richard V. Milani, Selim R. Krim, Daniel P. Morin

**Affiliations:** 1 Department of Cardiovascular Diseases, John Ochsner Heart and Vascular Institute, 1514 Jefferson Highway, New Orleans, LA 70121-2483 USA; 2 Ochsner Clinical School, Queensland University School of Medicine, New Orleans, LA USA

**Keywords:** Left ventricular dysfunction, Ischemic cardiomyopathy, Cardiac PET, Coronary flow capacity, Myocardial blood flow

## Abstract

**Purpose of Review:**

Cardiomyopathy with underlying left ventricular (LV) dysfunction is a heterogenous group of disorders that may be present with, and/or secondary to, coronary artery disease (CAD). The purpose of this review is to demonstrate, via case illustrations, the benefits offered by cardiac positron-emission tomography (PET) stress testing with coronary flow capacity (CFC) in the evaluation and treatment of patients with left ventricular (LV) dysfunction and CAD.

**Recent Findings:**

CFC, a metric that is increasing in prominence, represents the integration of several absolute perfusion metrics into clinical strata of CAD severity. Our prior work has demonstrated improvement in regional perfusion metrics as a result of revascularization to territories with severe reduction in CFC. Conversely, when CFC is adequate, there is no change in regional perfusion metrics following revascularization, despite angiographically severe stenosis. Furthermore, Gould et al. demonstrated decreased rates of myocardial infarction and death following revascularization of myocardium with severely reduced CFC, with no clinical benefit observed following revascularization of patients with preserved CFC. In a series of cases, we present pre-revascularization and post-revascularization PET scans with perfusion metrics in patients with LV dysfunction and CAD. In these examples, we demonstrate improvement in LV function and perfusion metrics following revascularization only in cases where baseline CFC is severely reduced.

**Summary:**

PET with CFC offers unique guidance regarding revascularization in patients with reduced LV function and CAD.

## Introduction

Left ventricular (LV) dysfunction can occur in the presence or absence of obstructive epicardial coronary artery disease (CAD). In the setting of LV dysfunction and obstructive CAD, in addition to medical therapies, current guidelines recommend coronary revascularization (level of evidence 2B) [1]. In the Surgical Treatment for Ischemic Heart Failure (STICH) trial, patients with an LV ejection fraction less than 35% were randomized to CABG versus medical therapy, provided “CAD suitable for CABG” was present and LM stenosis ≥ 50% was excluded. Cardiac death was less frequently observed in patients who underwent CABG rather than medical therapy after 10 years of follow-up [2].

The basic tenets from STICH, and the current presumption within published Guidelines, assume a universally causal relationship between CAD and LV dysfunction, as opposed to two distinct coexisting common pathologies: a primary cardiomyopathy and concomitant CAD. Classification of cardiomyopathies has proven to be complex given the myriad of potential and possibly overlapping underlying pathologic processes [3, 4]. In fact, there is no consensus on the definition of the term “ischemic cardiomyopathy” [3]. Furthermore, our ability to distinguish between a primary cardiomyopathy with concomitant CAD versus LV dysfunction resulting from epicardial CAD is imperfect yet crucial given the significant differences in optimal treatment.

Cardiac positron-emission tomography (PET) with quantitative perfusion is considered the non-invasive gold standard for measurement of MBF and coronary flow capacity (CFC) [5, 6^•^]. Data have demonstrated improvement in regional and global MBF, in addition to decreased morbidity and mortality, following revascularization procedures when baseline CFC was severely reduced. Furthermore, data also have demonstrated no improvement in MBF or mortality rate following revascularization of regions with CAD but preserved CFC [7••, 8^••^]. Hence, PET with quantitative perfusion and CFC potentially offers an improved methodology for selecting which patients with LV dysfunction and CAD would benefit from revascularization.

This article briefly reviews the status quo, as well as the principles of CFC, and how it differs from simple PET with absolute flow metrics in the typical 17-segment model. Finally, through a series of cases, we illustrate the principles for using PET with CFC to select patients with LV dysfunction for revascularization.

## The Status Quo

As noted above, current ACCF/AHA guidelines support revascularization for patients with LV dysfunction and multivessel stable CAD. The most common metric used for revascularization decisions and supported within current guidelines is visually assessed percent stenosis as seen on coronary angiography [9]. However, percent stenosis can be subjective, with significant amount of intra-observer variability. Furthermore, although this may be counterintuitive, percent stenosis is not predictive of adequate myocardial blood flow [5, 10]. There now is a large body of evidence suggesting that physiologic metrics, such as FFR, MBF, or coronary flow reserve (CFR), should replace percent stenosis for revascularization decisions. Despite these findings, angiographic percent stenosis remains the most common determinant of whether revascularization is pursued [11]. Although stress testing has taken a more prominent position in revascularization decisions, underlying LV dysfunction reduces per-vessel diagnostic accuracy using conventional stress modalities such as stress echocardiography and single photon emission computed tomography (SPECT) [12–14]. However, with its superior spatial resolution, attenuation correction, and physiologic correlation, PET stress testing with coronary flow capacity offers a new paradigm for assessing CAD in patients with LV dysfunction.

## Coronary Flow Capacity

Coronary flow capacity (CFC) is a novel metric first introduced by Johnson and Gould in 2012 [6•]. CFC was developed in an effort to simplify interpretation of the complex and numerous quantitative PET datasets by integrating several perfusion metrics into a single spectrum of clinical CAD severity [6•]. The methodologic specifics regarding CFC can be found within several references [6•, 8••, 15]. In brief, CFC is an integration of resting MBF, stress MBF, and CFR on a pixel-by-pixel basis into strata of pathophysiologic severity. Each PET scan produces a total of 1344 pixels that are presented in a color-coded map that provides perfusion distributions that are directly referable to how the epicardial vessels may appear on angiography. This method provides a granular assessment of regional coronary physiology that is not confined to nonbiologic external boundaries such as those found on polar maps. “Standard” methods of displaying MBF data using a 17-segment model within polar maps are spatially distorted and impose assumed arterial distributions. Further limitations of the “standard” 17-segment flow models have been discussed elsewhere [8••, 15].

Outcome data have demonstrated a 54% reduction in death and MI in patients with baseline severe reduction in CFC (i.e., the “blue” region on CFC maps) who received revascularization within 90 days of PET scanning compared with patients who had areas of severely reduced CFC but did not receive revascularization within 90 days [8••]. The proposed mechanism for enhanced survival is improvement in absolute flow as a result of revascularization in patients with “blue” CFC. While this mechanism may seem intuitively obvious, the explanation is nuanced. In our laboratory, we measured absolute perfusion metrics (MBF and CFC) in patients before and within 90 days after angiography. There was no requirement to base revascularization on the results of the PET scan’s relative perfusion images, and CFC information was not provided to the physicians performing angiography or revascularization. Thus, revascularization was based on “standard” practice which mostly equated to visual assessment of coronary stenosis. We concluded that mean absolute flow metrics improved ~60% in regions that had baseline “blue” (severe reduction) CFC and underwent revascularization. However, regions that were revascularized but were “non-blue” (i.e., no severe reduction in CFC) on PET imaging did not show improvement in perfusion metrics. Approximately 50% of the study group received revascularization that was concordant with “blue” regions. Of the remaining 50%, half received revascularization only to territories without severely reduced CFC [7••]. We concluded that revascularization improved MBF only when the regions undergoing revascularization were “blue” on pre-revascularization CFC maps. Visual assessment of CAD by percent stenosis did not correlate well with myocardial regions demonstrating severely reduced CFC. Furthermore, through multivariable regression analysis, we concluded that “blue” CFC was the only metric to predict improvement in MBF as a result of revascularization. These findings provide a mechanism and mirror outcome data from Gould et al. who recently demonstrated that size-dependent severe CFC was strongly correlated with high mortality that was reduced with revascularization, a finding not seen for global determinants such as CFR or sMBF [16••].

## Illustrative Cases

Each case illustrates one or more different principles to provide guidance for patients with cardiomyopathy and CAD. For each case, pre-revascularization and post-revascularization PET images as well as a graph demonstrating the percent change in absolute flow metrics are displayed. Day-to-day variability of perfusion metrics is ~20% and represented by the horizontal black lines on the graphs [17]. Perfusion metrics falling outside of the 20% range is secondary to revascularization procedures.

### Case 1/Fig. 1

The patient is a 50-year-old man with hypertension (HTN) and diabetes (DM). He was found to have LV dysfunction on echocardiography, with LVEF 25%. Pre-revascularization PET stress testing demonstrated a resting defect in the inferolateral wall (1st row). On stress (2nd row), there is a large and severe stress-induced confluent relative perfusion defect in all 3 vascular territories (blue regions) comprising 53% of the LV myocardium. CFC maps of integrated perfusion metrics (3rd row) demonstrate severely reduced flow capacity (“blue” regions) in 63% of the myocardium in all 3 major vascular territories. These findings portend high risk for death or MI and indicate that revascularization in all three vascular territories is appropriate. The patient underwent 3-vessel CABG (left internal mammary artery (LIMA)) to the left anterior descending artery (LAD), saphenous venous graft (SVG) to posterolateral branch (PLB), and SVG to the first obtuse marginal branch (OM1). The posterior descending artery was a poor operative target and did not undergo bypass grafting. Repeat PET stress testing, performed within 90 days of CABG as part of a research protocol, demonstrated marked improvement but not normalization of the relative perfusion images (1st and 2nd rows). On CFC maps (3rd row), there is marked reduction of the “blue” region following revascularization, though there remains severe reduction of CFC in the posterior descending artery (PDA) distribution (which was not revascularized). Absolute rest and stress MBF improved in all territories that were “blue” and underwent revascularization. Regional and whole-heart average stress MBF increased ~50% which is well above normal day-to-day variability. Post-operative LVEF was 40%, and LVEF 4 years after CABG was 50%. This case represents severe multivessel disease causing LV dysfunction. LV function improved following revascularization as a result of increased MBF and improvement in CFC.

**Fig. 1:**
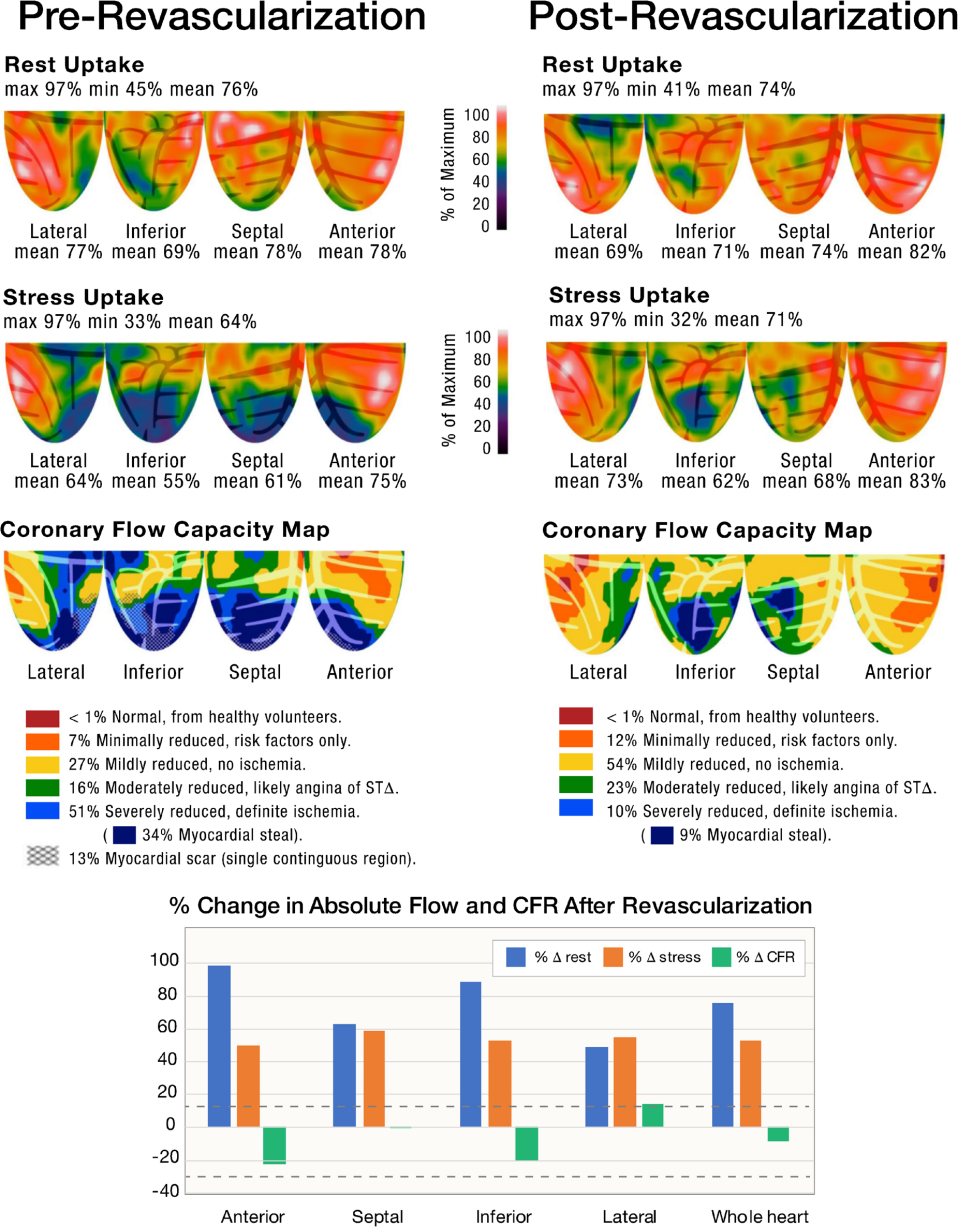
Pre-revascularization and post-revascularization rest and stress relative perfusion images (1st and 2nd rows, respectively) and coronary flow capacity maps (3rd row) of a patient with cardiomyopathy and associated perfusion abnormalities secondary to severe CAD. The patient history and revascularization specifics are located within the text. The graphs demonstrate the percent change from the baseline of regional resting MBF (*blue bars*), stress MBF (*orange bars*) and CFR (*gray bars*). The *black horizontal lines* denote 20% day-to-day normal variability of absolute perfusion. A change in absolute flow metrics outside of the *black horizontal lines* likely represents procedural or medical therapy influence on absolute perfusion. There is marked reduction of the “blue” regions in revascularized territories. Absolute rest and stress MBF improved in all territories that were “blue” and underwent revascularization. Regional and whole-heart average stress MBF increased ~50% which is well above normal day-to-day variability. LV function improved following revascularization as a result of increased MBF and improvement in CFC

### Case 2/Fig. 2

The patient is a 75-year-old woman with HTN, DM, hyperlipidemia, and CAD who underwent percutaneous intervention (PCI) of her LAD and right coronary artery (RCA) in the past. She presented with decompensated heart failure and was found to have an LVEF of 35% with global hypokinesis. Relative PET perfusion images demonstrate a very mild stress-induced defect in a wedge-shaped pattern in the basilar inferior wall (2nd row - mild yellow/green defect). This pattern is commonly seen in chronic occlusions of the RCA that are collateralized. CFC maps demonstrate severe reduction of CFC (blue zones) in all 3 coronary distributions. In addition, there is underlying microvascular dysfunction (yellow zones) with associated elevated resting MBF (not shown). This combination of multivessel obstructive disease and microvascular dysfunction result in near-normal relative perfusion scans, commonly termed “balanced ischemia.” The patient was found to have occlusion of the proximal RCA and high-grade stenosis of the mid LAD and mid LCX arteries and underwent successful PCI of all native vessels. Repeat PET stress testing, performed within 90 days of revascularization as part of a research protocol, demonstrated normalization of the relative perfusion images as well as marked improvement in CFC (no “blue”). Whole-heart stress MBF improved ~80%, and all revascularized regions demonstrated an increase in sMBF beyond expected 20% day-to-day variability. Subsequently, the LVEF improved to 60%. This case represents severe reduction of CFC despite relatively normal relative perfusion images due to “balanced ischemia,” illustrating the value of including CFC rather than relative imaging alone.

**Fig. 2:**
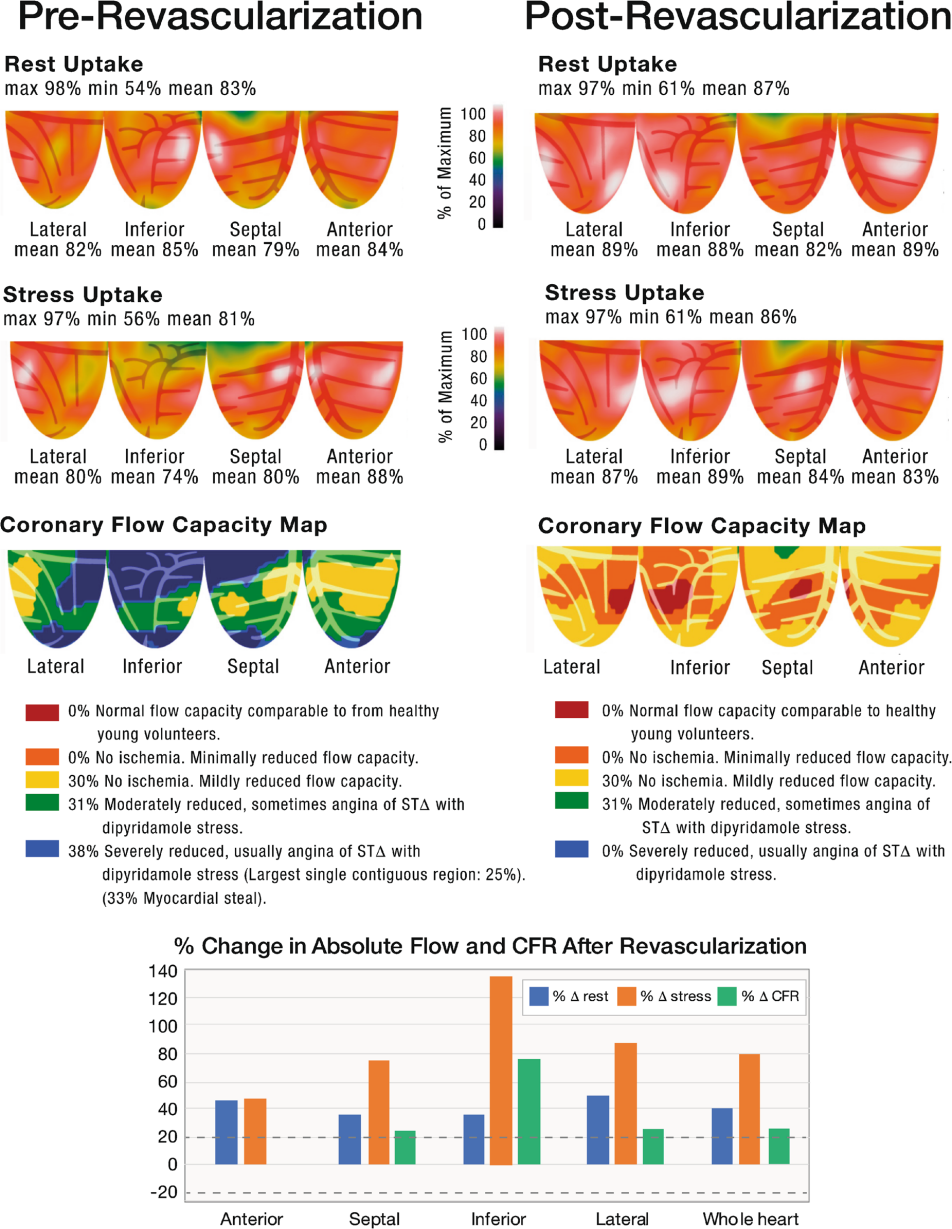
Pre-revascularization and post-revascularization rest and stress relative perfusion images (1st and 2nd rows, respectively) and coronary flow capacity maps (3rd row) of a patient with cardiomyopathy without significant perfusion abnormalities but with severely reduced “blue” CFC secondary to severe CAD. The patient history and revascularization specifics are located within the text. The graphs demonstrate the percent change from the baseline of regional resting MBF (*blue bars*), stress MBF (*orange bars*), and CFR (*gray bars*). The *black horizontal lines* denote 20% day-to-day normal variability of absolute perfusion. A change in absolute flow metrics outside of the *black horizontal lines* likely represents procedural or medical therapy influence on absolute perfusion. There is marked improvement in “blue” CFC with revascularization from 39 to 0% of the LV myocardium. Whole-heart stress MBF improved ~80% with regions improving ~40–120%. All revascularized regions demonstrated an increase in sMBF beyond expected 20% day-to-day variability

### Case 3/Fig. 3

The patient is a 43-year-old woman with HTN, DM, and end-stage renal disease on hemodialysis. As part of an evaluation prior to renal transplantation, her EF was found to be 35% with globally reduced LV function and regional akinesis of the lateral and inferoapical walls. PET stress demonstrated resting defects in the basilar lateral and inferoapical walls which became larger and more intense with stress. CFC maps demonstrate severe reduction (blue) in the OM and PDA territories. The LAD distribution, however, is free of regional perfusion abnormalities and shows preserved CFC (yellow/orange), which is consistent with the presence of CAD that is above ischemic thresholds, low risk, and unlikely to improve with revascularization. The patient subsequently underwent coronary angiography which demonstrated a left dominant circulation with occlusion of OM2 and the PDA. Both vessels were <1.5-mm vessels and poor targets for intervention. The LAD was noted to have a visually estimated proximal 90% stenosis. The patient underwent LIMA-to-LAD CABG without complications. Repeat PET stress testing, performed within 90 days of CABG as part of a research protocol, demonstrated nearly identical findings as the pre-revascularization PET without any significant change in absolute flow metrics nor CFC in the LAD distribution. All absolute PET flow metrics were within 20% of preoperative values (black horizontal lines on the graph). Approximately 1 year after CABG, the patient presented with an acute upper GI bleed and diabetic ketoacidosis. Repeat bedside echocardiography showed that the LVEF during this acute episode was 30%. She ultimately died as a result of these conditions. She did not have a repeat echocardiogram post-CABG with hemodynamic stability. This case represents cardiomyopathy due to a combination of ischemic CAD (LCx distribution) with an underlying primary “non-ischemic” cardiomyopathy (likely from DM) and visual overestimation of CAD severity (LAD territory). Revascularization of the physiologically uncompromised LAD region did not result in any improvement in myocardial flow.

**Fig. 3:**
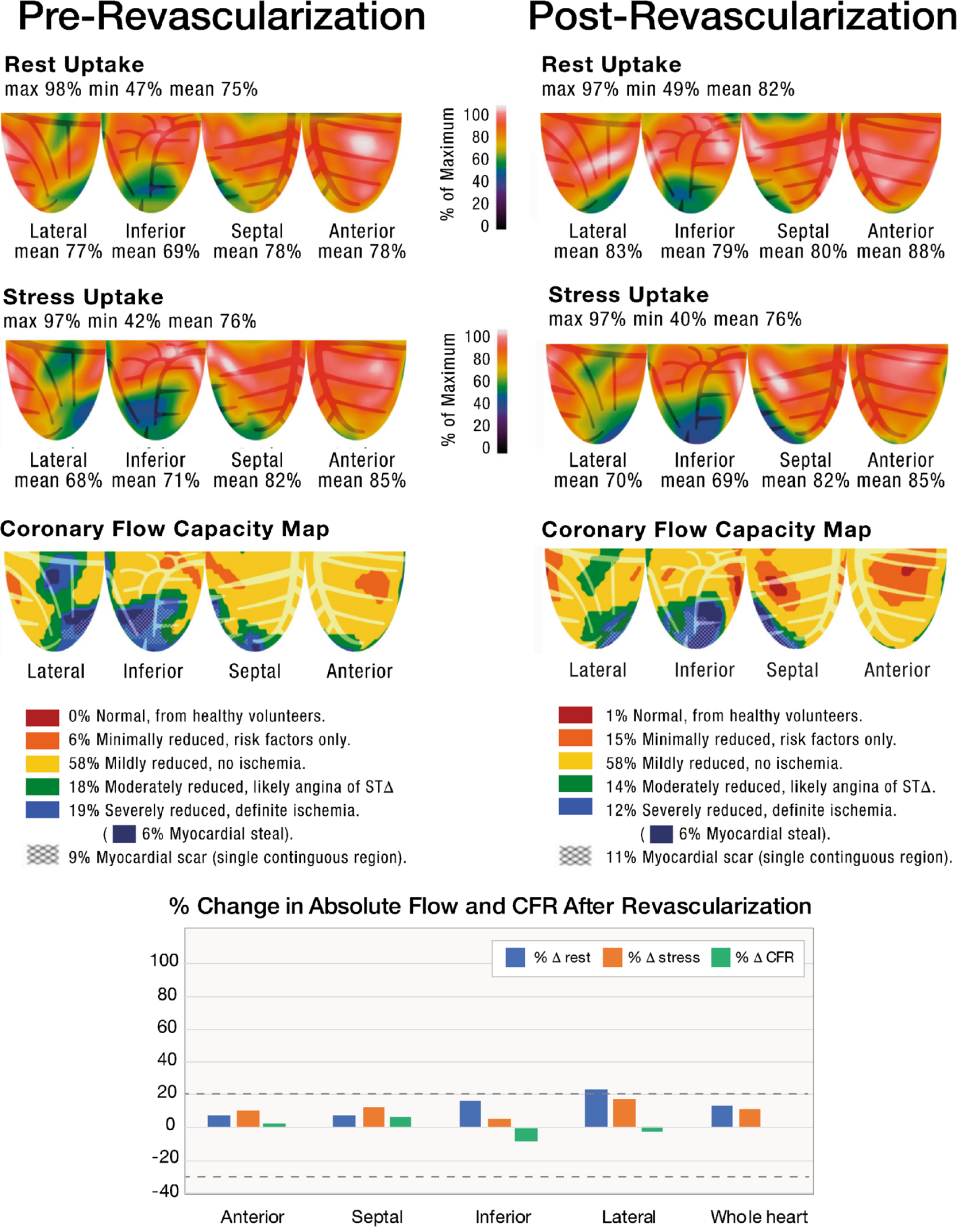
Pre-revascularization and post-revascularization rest and stress relative perfusion images (1st and 2nd rows, respectively) and coronary flow capacity maps (3rd row) of a patient with cardiomyopathy secondary to a combination of prior myocardial infarctions and a primary “non-ischemic” cardiomyopathy. The patient received a LIMA-to-LAD graft because of disease deemed significant based on visual assessment in the LAD and global cardiomyopathy. The patient history is located within the text. The graphs demonstrate the percent change from the baseline of regional resting MBF (*blue bars*), stress MBF (*orange bars*), and CFR (*gray bars*). The *black horizontal lines* denote 20% day-to-day normal variability of absolute perfusion. A change in absolute flow metrics outside of the *black horizontal lines* likely represents procedural or medical therapy influence on absolute perfusion. After revascularization, there was neither significant change in absolute flow metrics nor in CFC in the LAD distribution (revascularized territory) or the non-revascularized territory. All absolute PET flow metrics were within 20% of preoperative values

### Case 4/Fig. 4

The patient is a 70-year-old man who presented with syncope and was found on echocardiography to have global LV hypokinesis and an LV ejection fraction of 35–40%. On PET stress testing, relative perfusion images (1st and 2nd rows) demonstrated mild inferior wall heterogeneity on rest and stress. CFC maps (3rd row) demonstrated nearly near-normal (red) CFC in the majority of the myocardium. Despite the near-normal CFC, the patient underwent coronary angiography which demonstrated a 50% left main lesion with cross-sectional area of 4.4 mm^2^. The patient underwent 2 vessel CABG (LIMA-LAD and SVG-OM1) with an uncomplicated post-operative course. Following recovery from CABG, the patient experienced symptomatic ventricular tachycardia and required an implantable defibrillator. Repeat coronary angiograms demonstrated unchanged native coronary anatomy and patent coronary bypass grafts. Numerous echocardiograms demonstrated unchanged LV function with LVEF 35–40%. Repeat PET stress testing approximately 3 years after CABG demonstrated nearly identical findings as the pre-revascularization PET, with unchanged CFC within the revascularized territories. There was a slight decrease in CFC in the RCA territory, consistent with the progression of mild nonobstructive RCA disease or perhaps progression of a primary myocardial process. This case represents a primary “non-ischemic” cardiomyopathy with concomitant nonobstructive CAD. Revascularization that was performed because of the focal LM lesion neither improved LV function nor did it improve quantitative perfusion metrics.

**Fig. 4:**
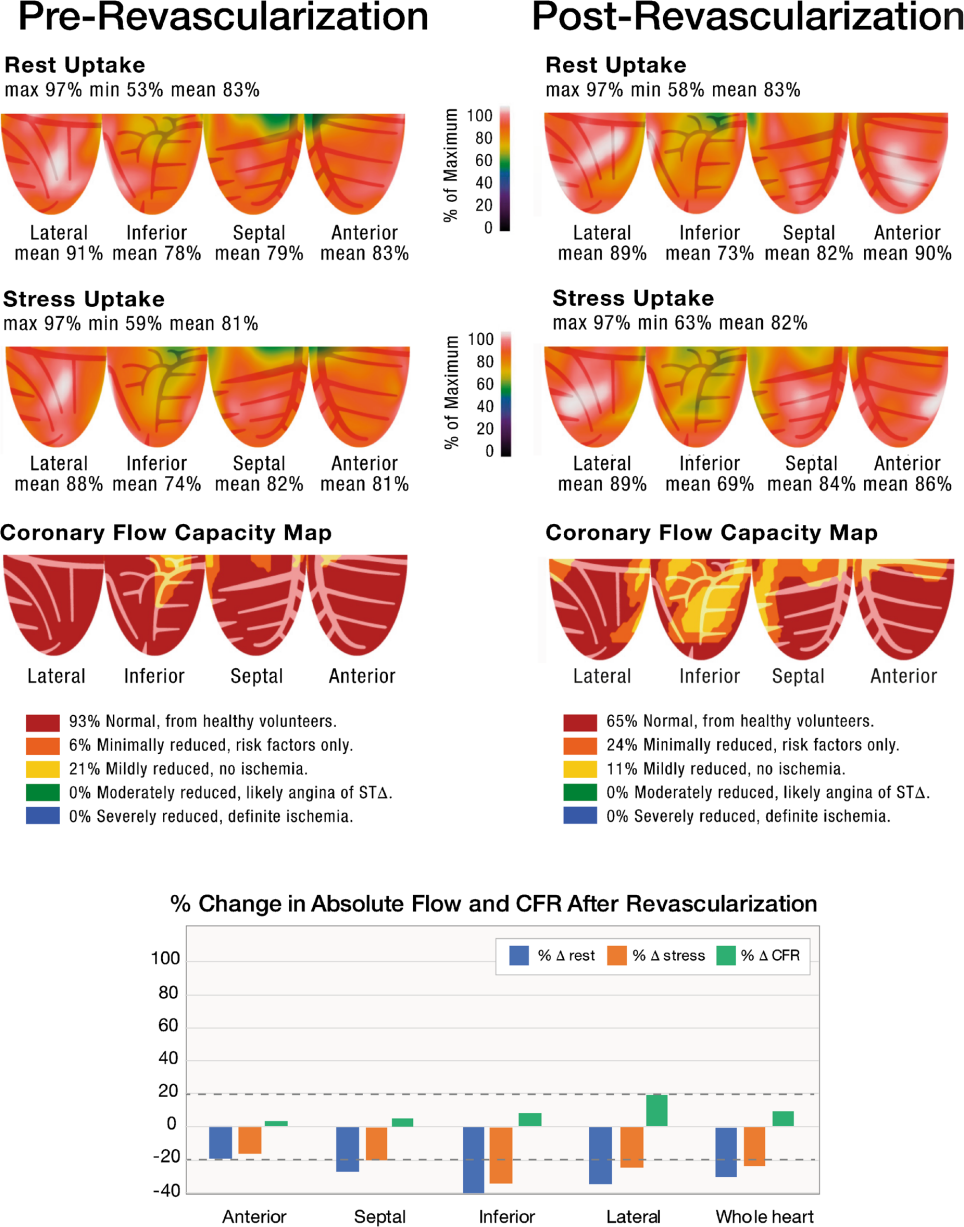
Pre-revascularization and post-revascularization rest and stress relative perfusion images (1st and 2nd rows, respectively) and coronary flow capacity maps (3rd row) of a patient with a primary “non-ischemic” cardiomyopathy with concomitant left main disease visually determined as “significant.” The patient received CABG (LIMA to the LAD and SVG to the OM). The patient history is located within the text. The graphs demonstrate the percent change from the baseline of regional resting MBF (*blue bars*), stress MBF (*orange bars*), and CFR (*gray bars*). The *black horizontal lines* denote 20% day-to-day normal variability of absolute perfusion. A change in absolute flow metrics outside of the *black horizontal lines* likely represents procedural or medical therapy influence on absolute perfusion. Repeat PET stress testing approximately 3 years after CABG demonstrated nearly identical findings as the pre-revascularization PET, with unchanged CFC within the revascularized territories (anterior, septal, and lateral walls). There was a slight decrease in CFC in the RCA territory, consistent with progression of mild nonobstructive RCA disease or perhaps progression of a primary myocardial process

## Conclusions

Cardiac positron-emission tomography with assessment of coronary flow capacity offers unique insights in patients with LV dysfunction and CAD and has the potential to improve revascularization decision-making in these patients.
